# Supplementary material for: Accurate and sensitive mutational signature analysis with MuSiCal
Source: Nat Genet. 2024 Feb 15;56(3):541–52. doi: 10.1038/s41588-024-01659-0 (PMC10937379; doi:10.1038/s41588-024-01659-0)
Supplement: Supplementary file 2 — Reporting Summary [file 41588_2024_1659_MOESM2_ESM.pdf]

nature portfolio

Corresponding author(s): Peter J. Park

Last updated by author(s): Dec 7, 2023

Reporting Summary

Nature Portfolio wishes to improve the reproducibility of the work that we publish. This form provides structure for consistency and transparency in reporting. For further information on Nature Portfolio policies, see our Editorial Policies and the Editorial Policy Checklist.

Statistics

For all statistical analyses, confirm that the following items are present in the figure legend, table legend, main text, or Methods section.

n/a	Confirmed	
☐	☒	The exact sample size (*n*) for each experimental group/condition, given as a discrete number and unit of measurement
☐	☒	A statement on whether measurements were taken from distinct samples or whether the same sample was measured repeatedly
☐	☒	The statistical test(s) used AND whether they are one- or two-sided<br>*Only common tests should be described solely by name; describe more complex techniques in the Methods section.*
☐	☒	A description of all covariates tested
☐	☒	A description of any assumptions or corrections, such as tests of normality and adjustment for multiple comparisons
☐	☒	A full description of the statistical parameters including central tendency (e.g. means) or other basic estimates (e.g. regression coefficient) AND variation (e.g. standard deviation) or associated estimates of uncertainty (e.g. confidence intervals)
☐	☒	For null hypothesis testing, the test statistic (e.g. *F*, *t*, *r*) with confidence intervals, effect sizes, degrees of freedom and *P* value noted<br>*Give P values as exact values whenever suitable.*
☒	☐	For Bayesian analysis, information on the choice of priors and Markov chain Monte Carlo settings
☐	☒	For hierarchical and complex designs, identification of the appropriate level for tests and full reporting of outcomes
☐	☒	Estimates of effect sizes (e.g. Cohen's *d*, Pearson's *r*), indicating how they were calculated

Our web collection on statistics for biologists contains articles on many of the points above.

Software and code

Policy information about availability of computer code

Data collection	No software was used for data collection.
Data analysis	MuSiCal is implemented in Python and available at https://github.com/parklab/MuSiCal. SigProfilerExtractor (version 1.1.3) was downloaded from https://github.com/AlexandrovLab/SigProfilerExtractor. sigLASSO (version 1.1) was downloaded from https://github.com/gersteinlab/siglasso. signature.tools.lib (version 2.1.2) was downloaded from https://github.com/Nik-Zainal-Group/signature.tools.lib. SignatureAnalyzer (version 0.0.7) was downloaded from https://github.com/getzlab/SignatureAnalyzer. SigneR (version 1.22.0) was obtained from https://www.bioconductor.org/packages/release/bioc/html/signeR.html. Custom scripts and analysis notebooks for reproducing results in the paper are available on Zenodo: https://doi.org/10.5281/zenodo.10291569.

For manuscripts utilizing custom algorithms or software that are central to the research but not yet described in published literature, software must be made available to editors and reviewers. We strongly encourage code deposition in a community repository (e.g. GitHub). See the Nature Portfolio guidelines for submitting code & software for further information.

Data

Policy information about availability of data

All manuscripts must include a data availability statement. This statement should provide the following information, where applicable:

- Accession codes, unique identifiers, or web links for publicly available datasets
- A description of any restrictions on data availability
- For clinical datasets or third party data, please ensure that the statement adheres to our policy

PCAWG data, including mutation count matrices of SBSs and IDs, as well as exposure matrices, were downloaded from https://www.synapse.org/#!Synapse:syn11726601/files/. Additional PCAWG data were downloaded from https://dcc.icgc.org/releases/PCAWG. All PCAWG data used in this paper are in the open tier. COSMIC signatures were downloaded from https://cancer.sanger.ac.uk/signatures/. Source data, including MuSiCal-derived signature catalog from the PCAWG reanalysis, are provided in Supplementary Tables 1-4.

Research involving human participants, their data, or biological material

Policy information about studies with human participants or human data. See also policy information about sex, gender (identity/presentation), and sexual orientation and race, ethnicity and racism.

Reporting on sex and gender	*Use the terms sex (biological attribute) and gender (shaped by social and cultural circumstances) carefully in order to avoid confusing both terms. Indicate if findings apply to only one sex or gender; describe whether sex and gender were considered in study design; whether sex and/or gender was determined based on self-reporting or assigned and methods used. Provide in the source data disaggregated sex and gender data, where this information has been collected, and if consent has been obtained for sharing of individual-level data; provide overall numbers in this Reporting Summary. Please state if this information has not been collected. Report sex- and gender-based analyses where performed, justify reasons for lack of sex- and gender-based analysis.*
Reporting on race, ethnicity, or other socially relevant groupings	*Please specify the socially constructed or socially relevant categorization variable(s) used in your manuscript and explain why they were used. Please note that such variables should not be used as proxies for other socially constructed/relevant variables (for example, race or ethnicity should not be used as a proxy for socioeconomic status). Provide clear definitions of the relevant terms used, how they were provided (by the participants/respondents, the researchers, or third parties), and the method(s) used to classify people into the different categories (e.g. self-report, census or administrative data, social media data, etc.) Please provide details about how you controlled for confounding variables in your analyses.*
Population characteristics	Participants were recruited in each individual ICGC study and in TCGA. All these studies complied with the required ethical guidelines.
Recruitment	See above.
Ethics oversight	See publications of the corresponding ICGC, TCGA, and PCAWG projects.

Note that full information on the approval of the study protocol must also be provided in the manuscript.

Field-specific reporting

Please select the one below that is the best fit for your research. If you are not sure, read the appropriate sections before making your selection.

☒ Life sciences ☐ Behavioural & social sciences ☐ Ecological, evolutionary & environmental sciences

For a reference copy of the document with all sections, see nature.com/documents/nr-reporting-summary-flat.pdf

Life sciences study design

All studies must disclose on these points even when the disclosure is negative.

Sample size	This study was designed to be a retrospective analysis of previously published data. No statistical method was used to predetermine sample size.
Data exclusions	We only considered sequencing data passing the Quality & Control criteria established by the Pan-Cancer Analysis of Whole Genomes (PCAWG) project. The quality control of this dataset is discussed in their corresponding publication.
Replication	No replication was performed since the study was a retrospective analysis of existing datasets.
Randomization	No randomization was performed since the study was observational.
Blinding	The Investigators were not blinded to allocation during experiments and outcome assessment since the study was observational.

Reporting for specific materials, systems and methods

We require information from authors about some types of materials, experimental systems and methods used in many studies. Here, indicate whether each material, system or method listed is relevant to your study. If you are not sure if a list item applies to your research, read the appropriate section before selecting a response.

Materials & experimental systems

n/a	Involved in the study
☒	☐ Antibodies
☒	☐ Eukaryotic cell lines
☒	☐ Palaeontology and archaeology
☒	☐ Animals and other organisms
☒	☐ Clinical data
☒	☐ Dual use research of concern
☒	☐ Plants

Methods

n/a	Involved in the study
☒	☐ ChIP-seq
☒	☐ Flow cytometry
☒	☐ MRI-based neuroimaging
