# Supplementary material for: Accurate and sensitive mutational signature analysis with MuSiCal
Source: Nat Genet. 2024 Feb 15;56(3):541–52. doi: 10.1038/s41588-024-01659-0 (PMC10937379; doi:10.1038/s41588-024-01659-0)
Supplement: Supplementary file 4 — Simulated examples as in Fig. 3a, but for other tumor types. [file 41588_2024_1659_MOESM4_ESM.pdf]

Supplementary Data

In the Supplementary Data below, we show simulated examples as in Fig. 3a, but for the other 24 tumor types. Specifically, the true signatures were plotted in panel (a), where the percentages below signature names denote exposure strengths of corresponding signatures. *De novo* signatures extracted by MuSiCal were plotted in panel (b). *De novo* signatures extracted by SigProfilerExtractor were plotted in panel (c). The precision-recall curves (PRCs) for both methods were plotted in panel (d), with area under PRC (auPRC) annotated. The matching result corresponding to the largest achieved F1 score was shown in panels (e) and (f) for MuSiCal and SigProfilerExtractor, respectively. Tumor types were ordered as in Fig. 3b.

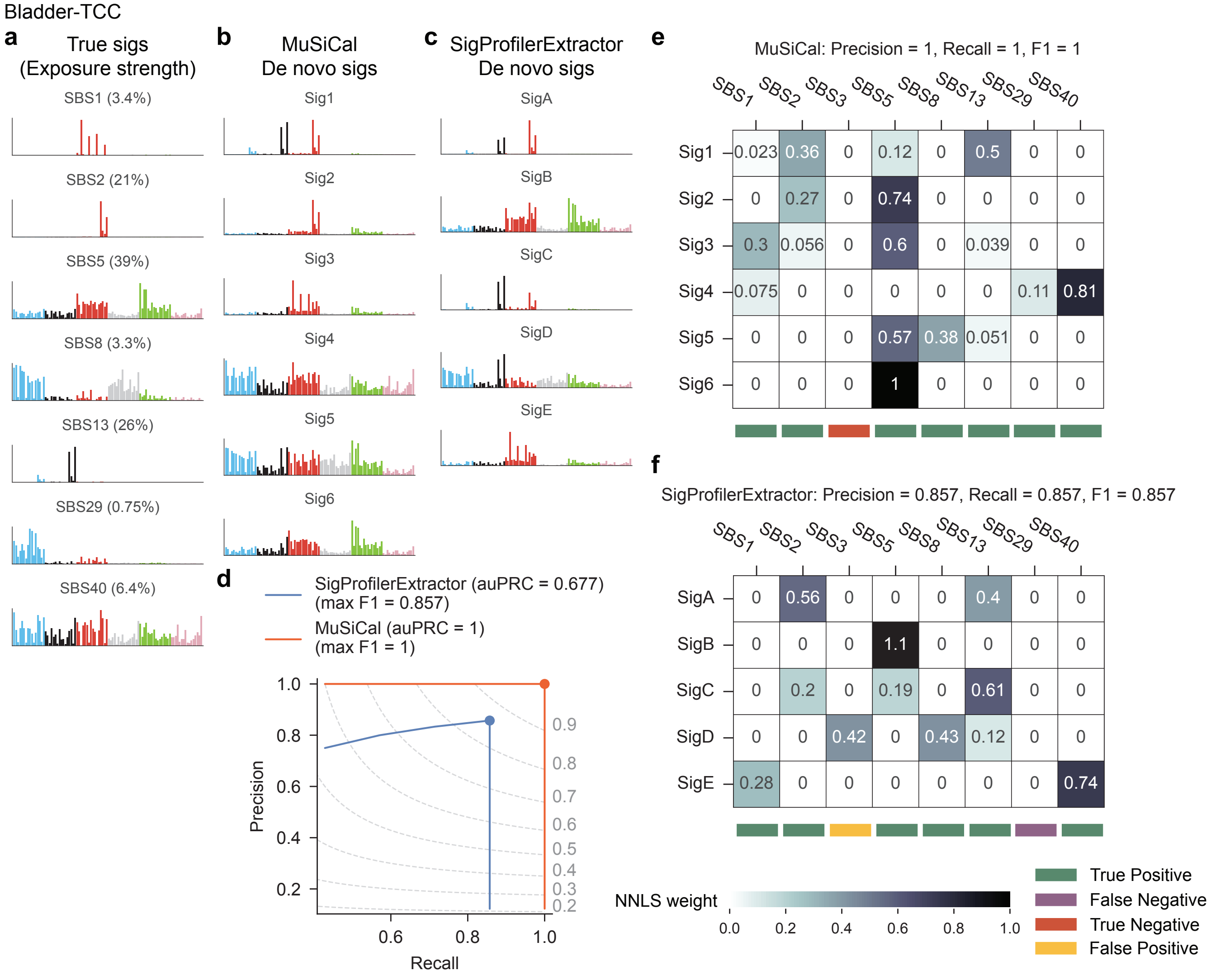

Supplementary Data Fig. 1 Same as Fig. 3a, but for Bladder-TCC.

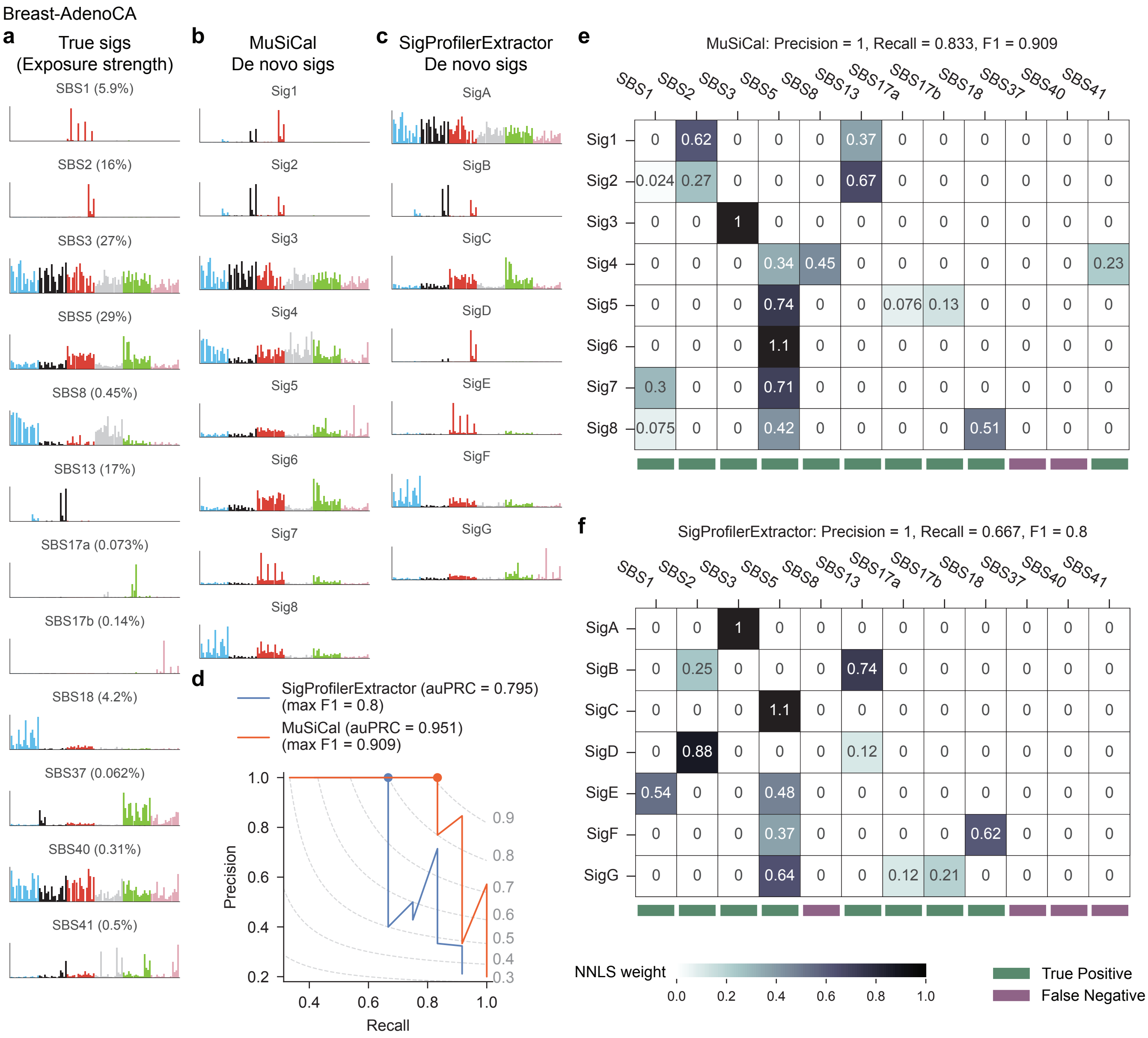

Supplementary Data Fig. 2 Same as Fig. 3a, but for Breast-AdenoCA.

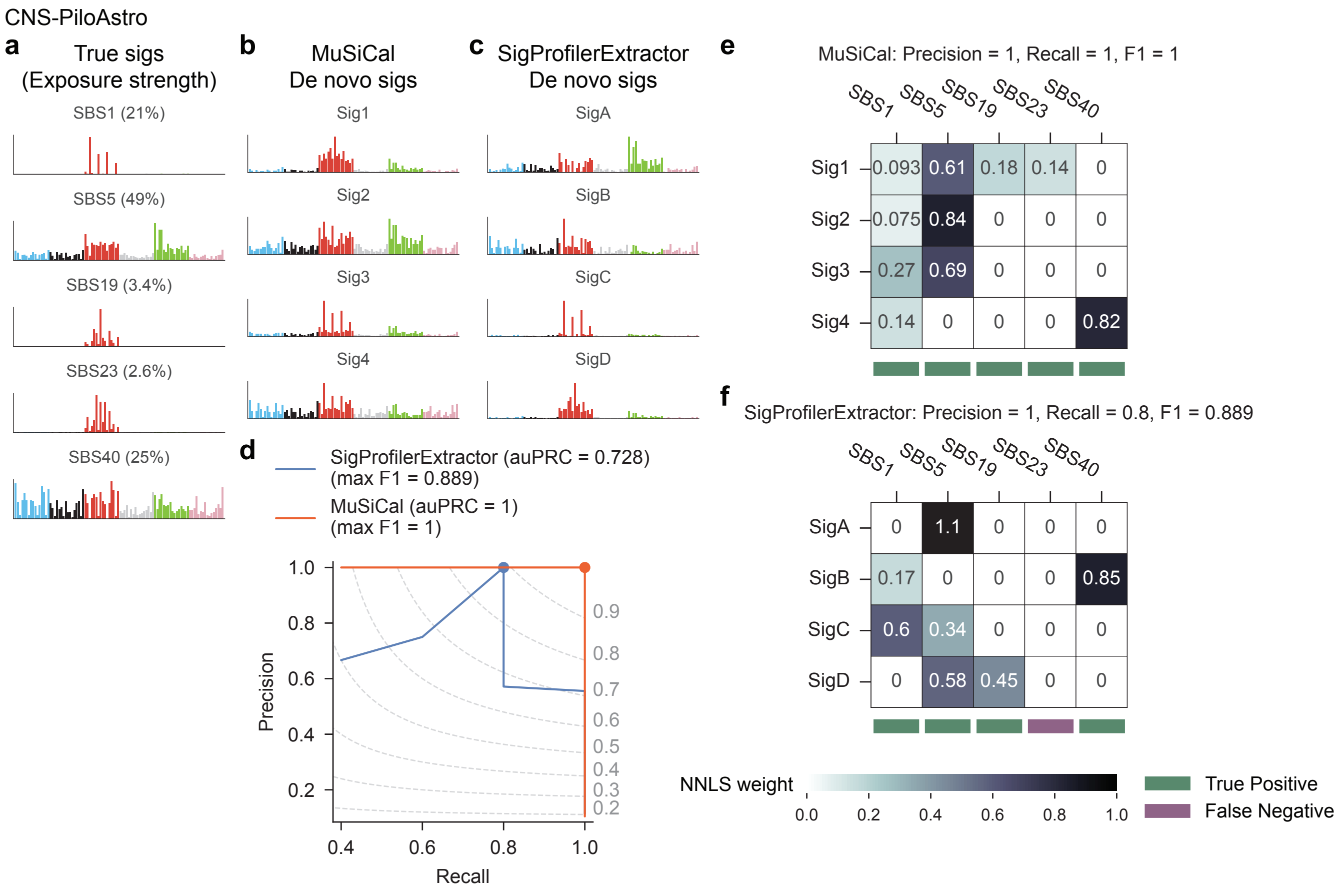

Supplementary Data Fig. 3 Same as Fig. 3a, but for CNS-PiloAstro.

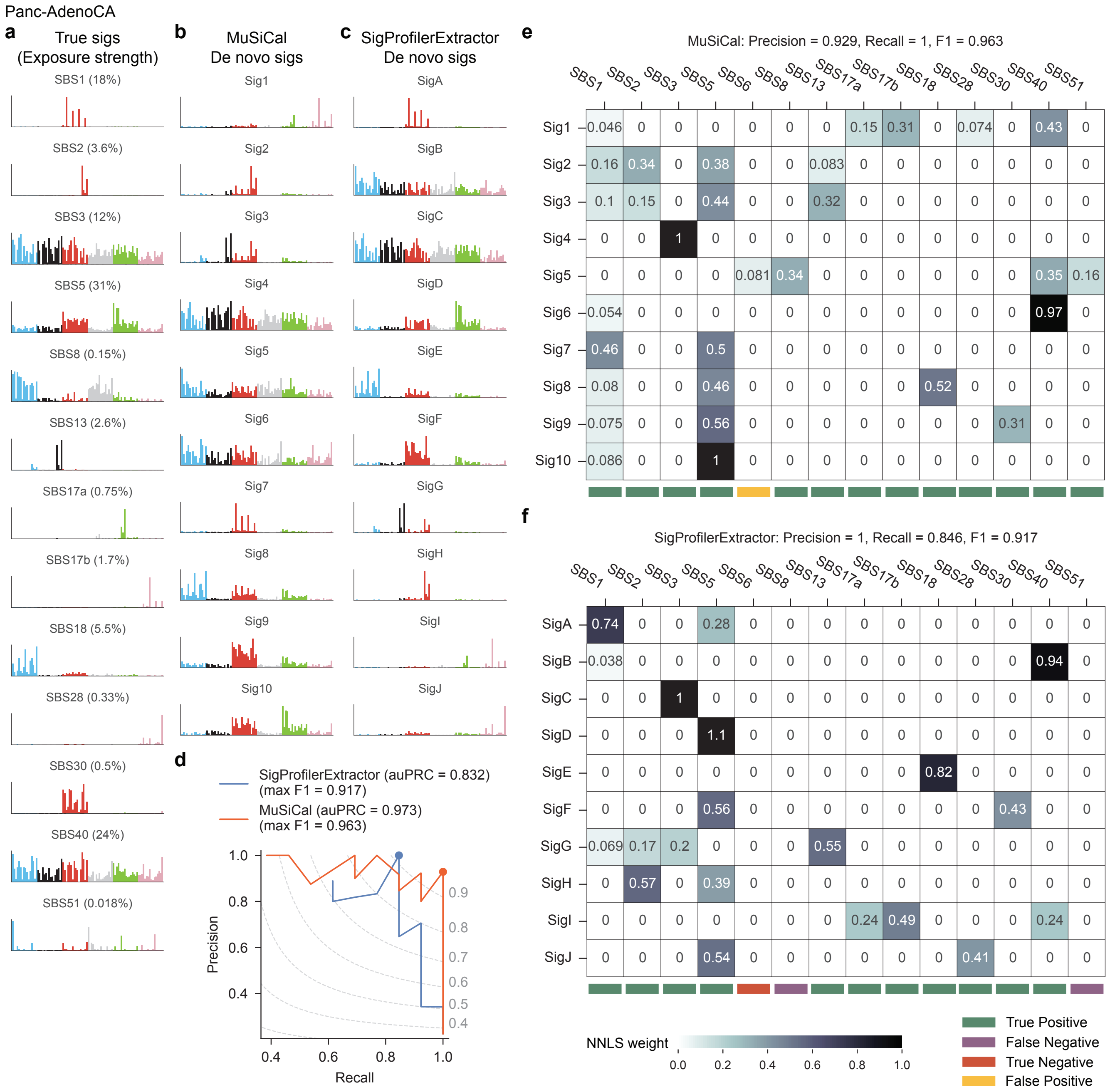

Supplementary Data Fig. 4 Same as Fig. 3a, but for Panc-AdenoCA.

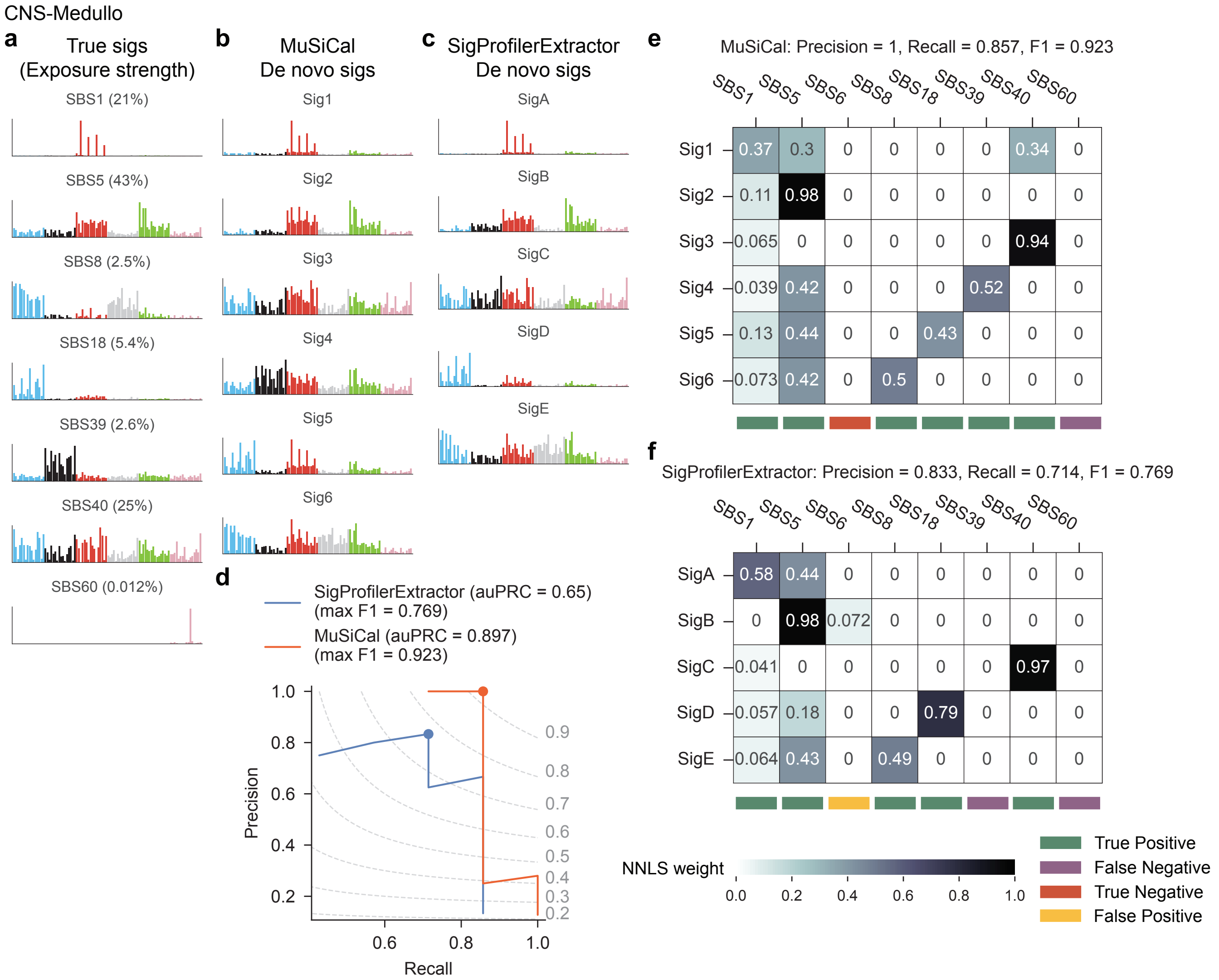

Supplementary Data Fig. 5 Same as Fig. 3a, but for CNS-Medullo.

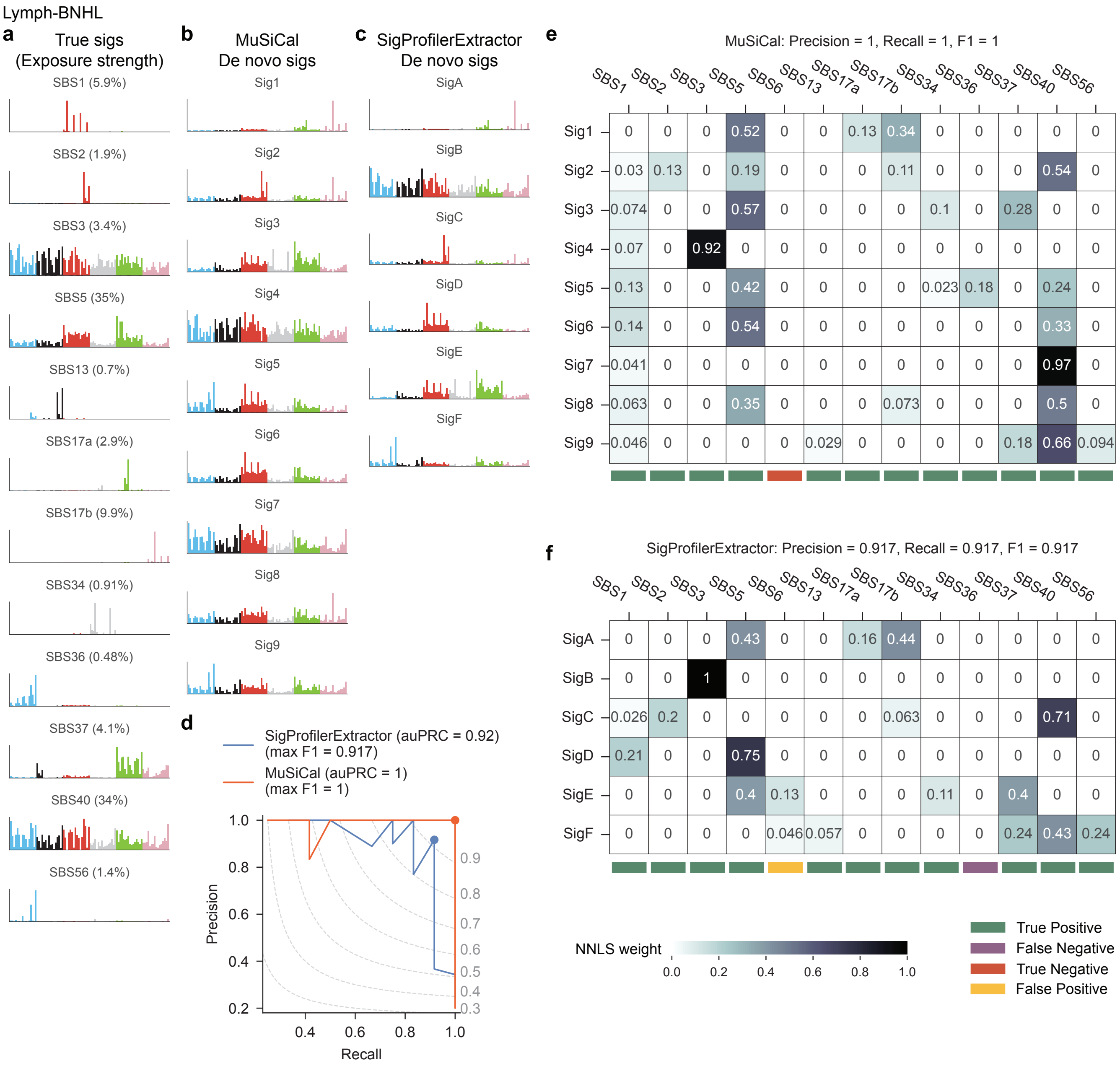

Supplementary Data Fig. 6 Same as Fig. 3a, but for Lymph-BNHL.

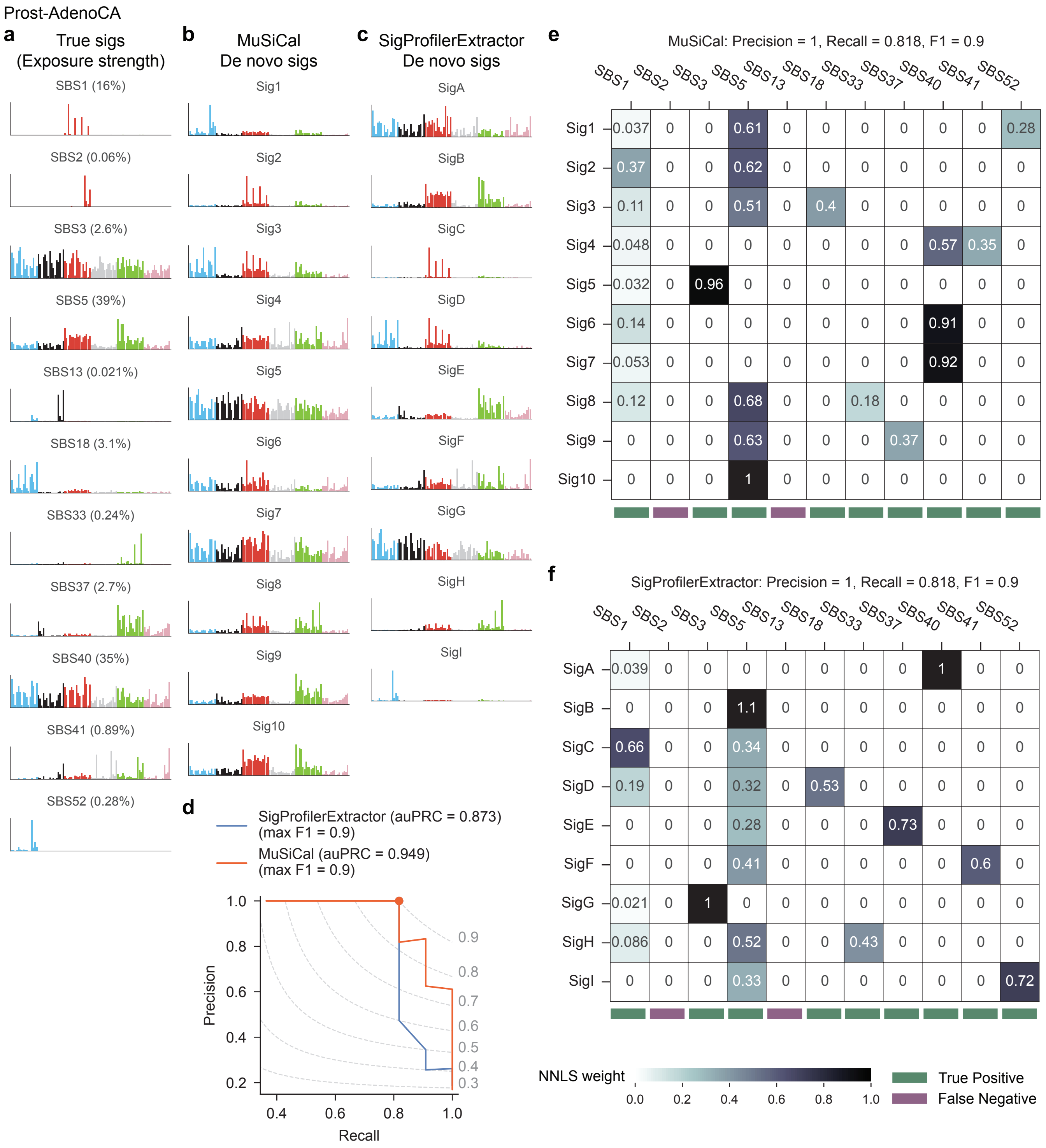

	SBS1	SBS2	SBS3	SBS5	SBS13	SBS18	SBS33	SBS37	SBS40	SBS41	SBS52
Sig1	0.037	0	0	0.61	0	0	0	0	0	0	0.28
Sig2	0.37	0	0	0.62	0	0	0	0	0	0	0
Sig3	0.11	0	0	0.51	0	0.4	0	0	0	0	0
Sig4	0.048	0	0	0	0	0	0	0	0.57	0.35	0
Sig5	0.032	0	0.96	0	0	0	0	0	0	0	0
Sig6	0.14	0	0	0	0	0	0	0	0.91	0	0
Sig7	0.053	0	0	0	0	0	0	0	0.92	0	0
Sig8	0.12	0	0	0.68	0	0	0.18	0	0	0	0
Sig9	0	0	0	0.63	0	0	0	0.37	0	0	0
Sig10	0	0	0	1	0	0	0	0	0	0	0

	SBS1	SBS2	SBS3	SBS5	SBS13	SBS18	SBS33	SBS37	SBS40	SBS41	SBS52
SigA	0.039	0	0	0	0	0	0	0	1	0	0
SigB	0	0	0	1.1	0	0	0	0	0	0	0
SigC	0.66	0	0	0.34	0	0	0	0	0	0	0
SigD	0.19	0	0	0.32	0	0.53	0	0	0	0	0
SigE	0	0	0	0.28	0	0	0	0.73	0	0	0
SigF	0	0	0	0.41	0	0	0	0	0	0.6	0
SigG	0.021	0	1	0	0	0	0	0	0	0	0
SigH	0.086	0	0	0.52	0	0	0.43	0	0	0	0
SigI	0	0	0	0.33	0	0	0	0	0	0	0.72

Supplementary Data Fig. 7 Same as Fig. 3a, but for Prost-AdenoCA.

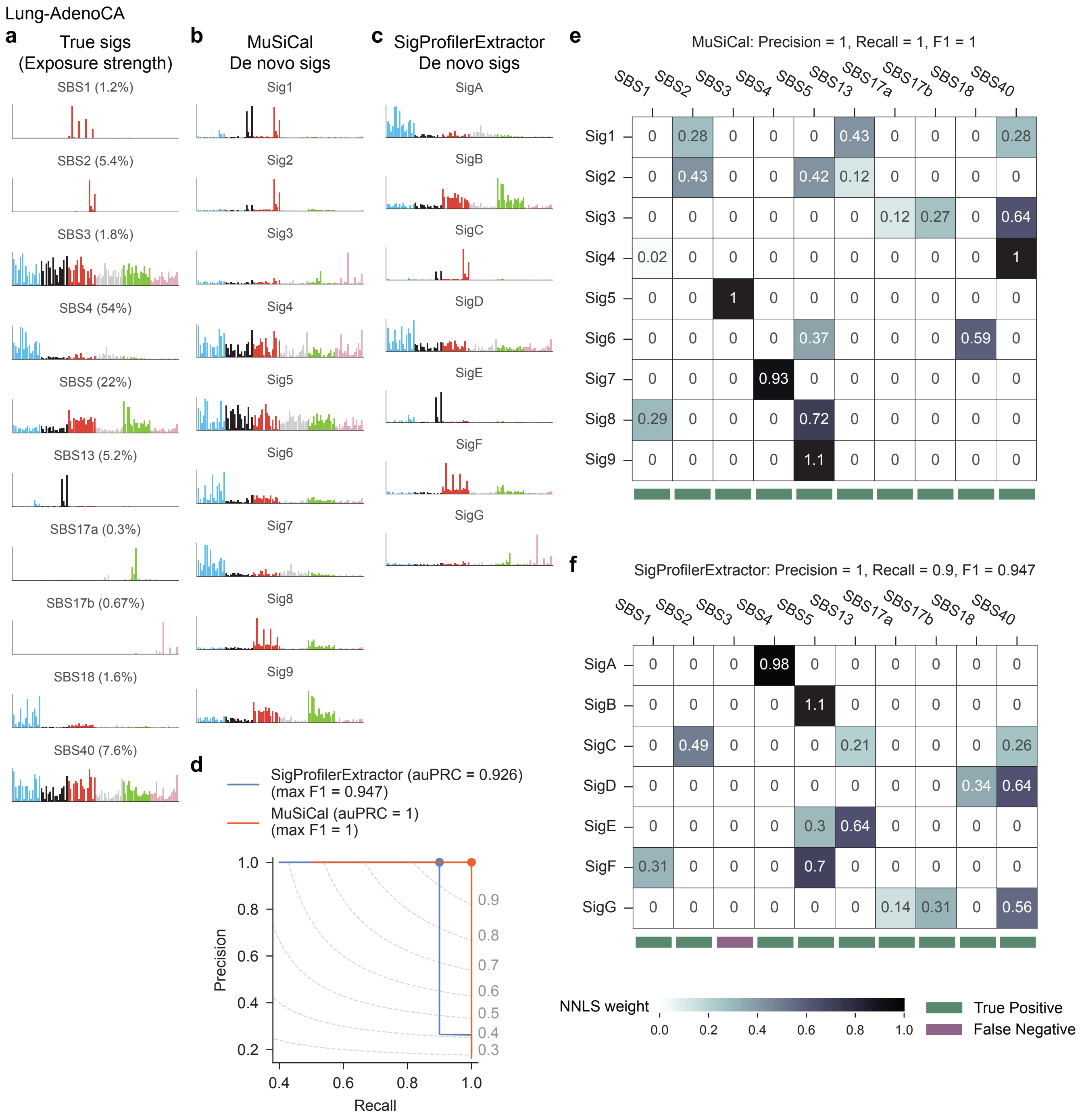

Supplementary Data Fig. 8 Same as Fig. 3a, but for Lung-AdenoCA.

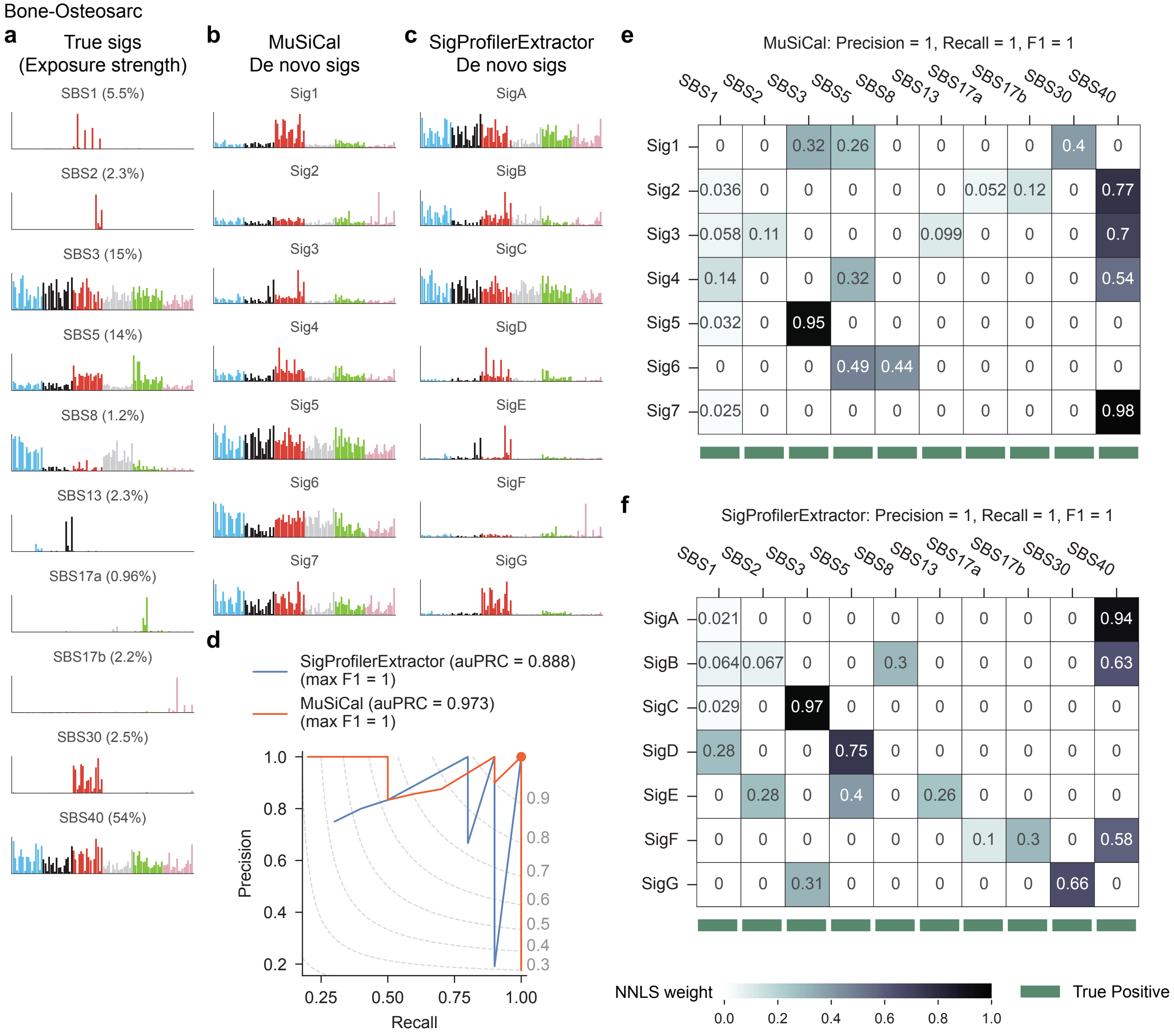

Supplementary Data Fig. 9 Same as Fig. 3a, but for Bone-Osteosarc.

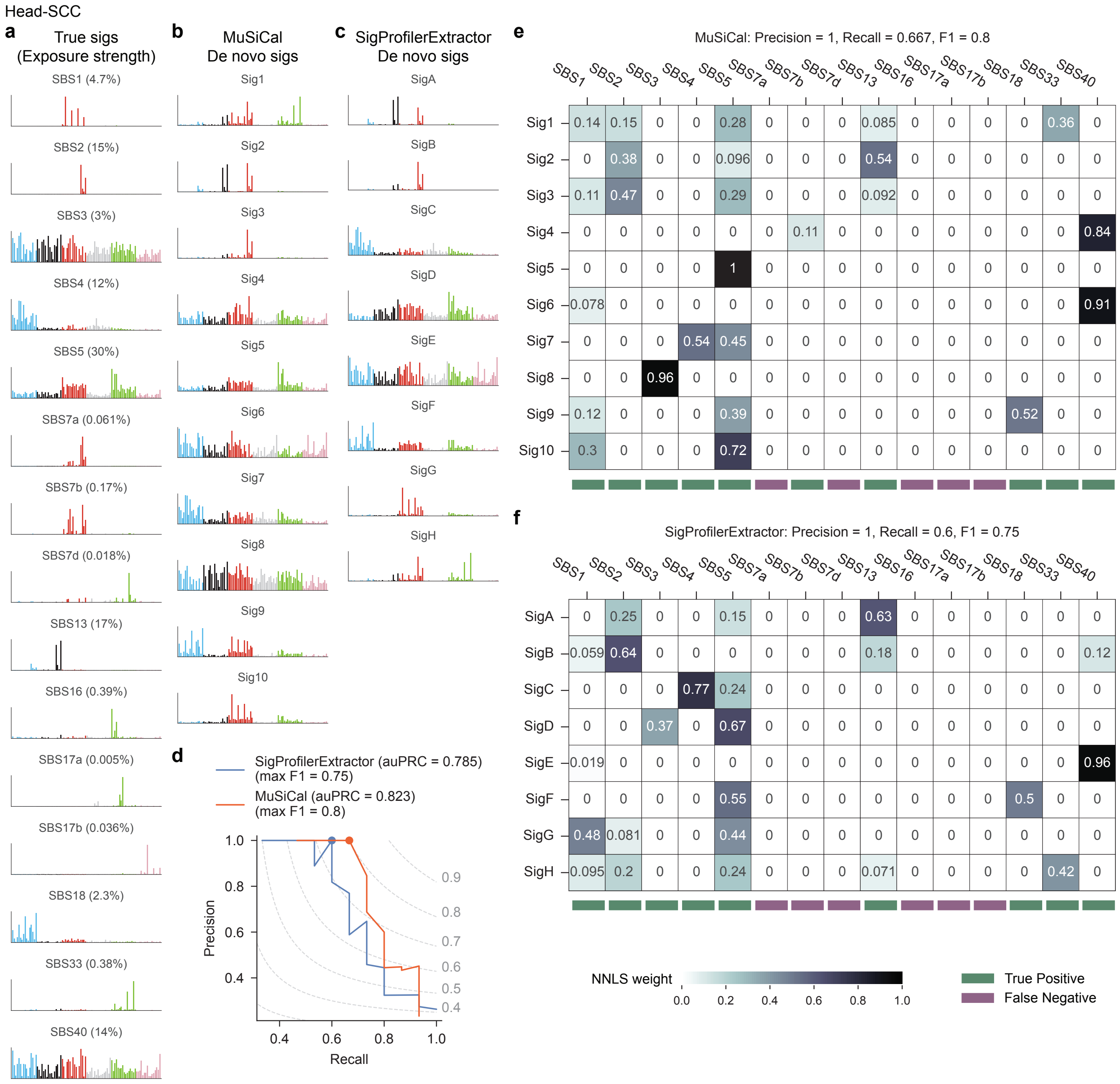

Supplementary Data Fig. 10 Same as Fig. 3a, but for Head-SCC.

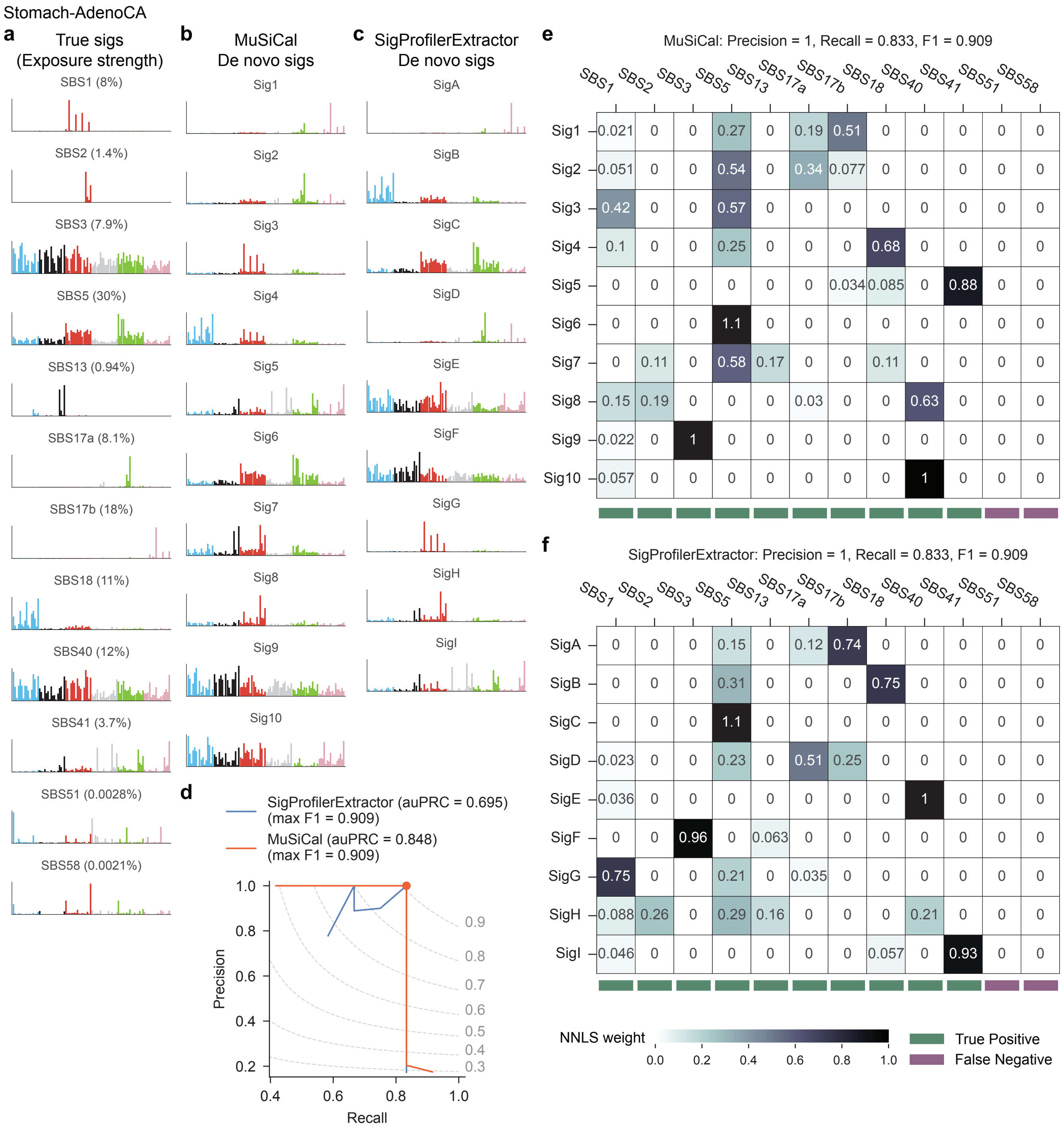

Supplementary Data Fig. 11 Same as Fig. 3a, but for Stomach-AdenoCA.

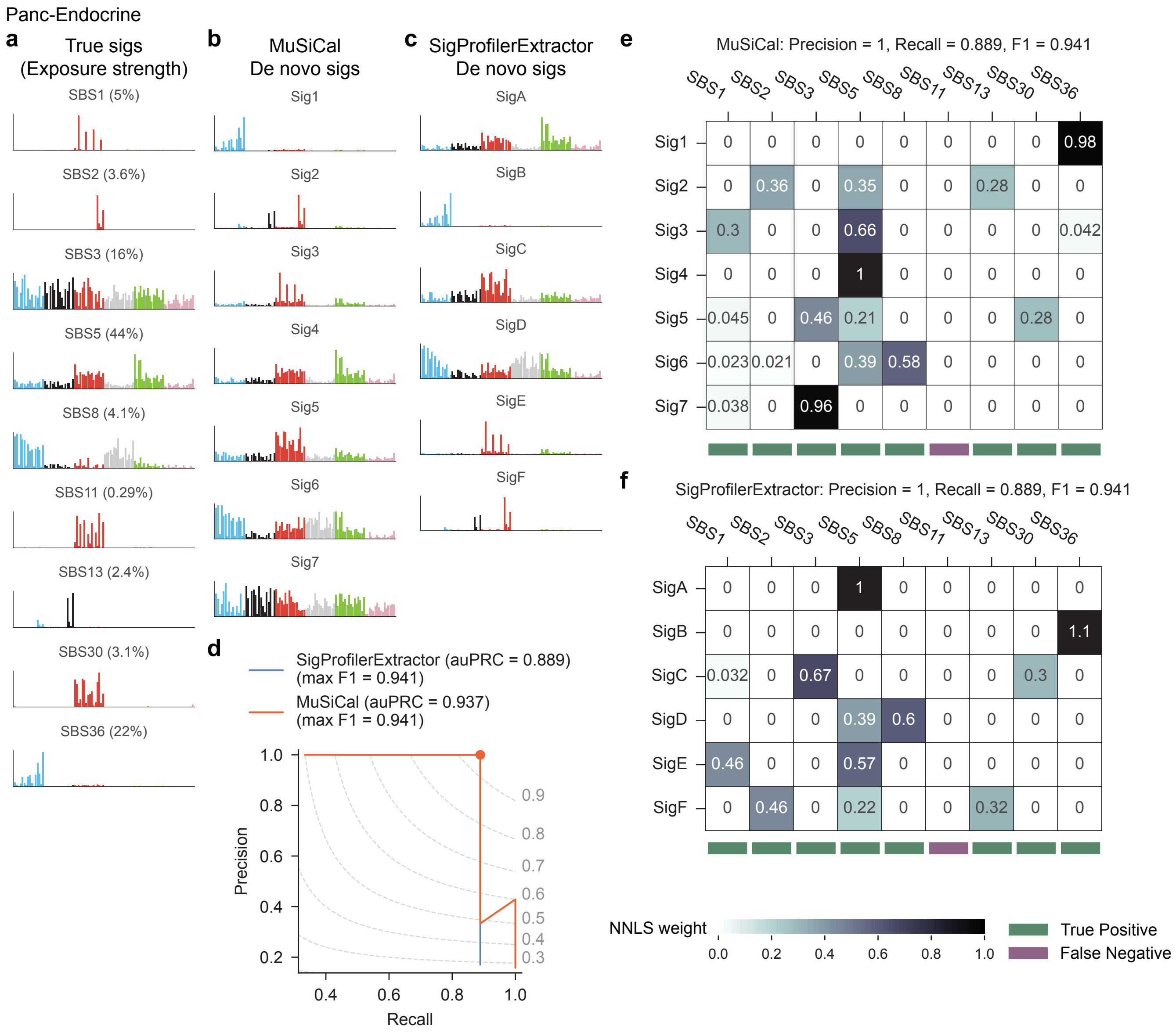

	SBS1	SBS2	SBS3	SBS5	SBS8	SBS11	SBS13	SBS30	SBS36
Sig1	0	0	0	0	0	0	0	0	0.98
Sig2	0	0.36	0	0.35	0	0	0.28	0	0
Sig3	0.3	0	0	0.66	0	0	0	0	0.042
Sig4	0	0	0	1	0	0	0	0	0
Sig5	0.045	0	0.46	0.21	0	0	0	0.28	0
Sig6	0.023	0.021	0	0.39	0.58	0	0	0	0
Sig7	0.038	0	0.96	0	0	0	0	0	0

	SBS1	SBS2	SBS3	SBS5	SBS8	SBS11	SBS13	SBS30	SBS36
SigA	0	0	0	1	0	0	0	0	0
SigB	0	0	0	0	0	0	0	0	1.1
SigC	0.032	0	0.67	0	0	0	0	0.3	0
SigD	0	0	0	0.39	0.6	0	0	0	0
SigE	0.46	0	0	0.57	0	0	0	0	0
SigF	0	0.46	0	0.22	0	0	0.32	0	0

Supplementary Data Fig. 12 Same as Fig. 3a, but for Panc-Endocrine.

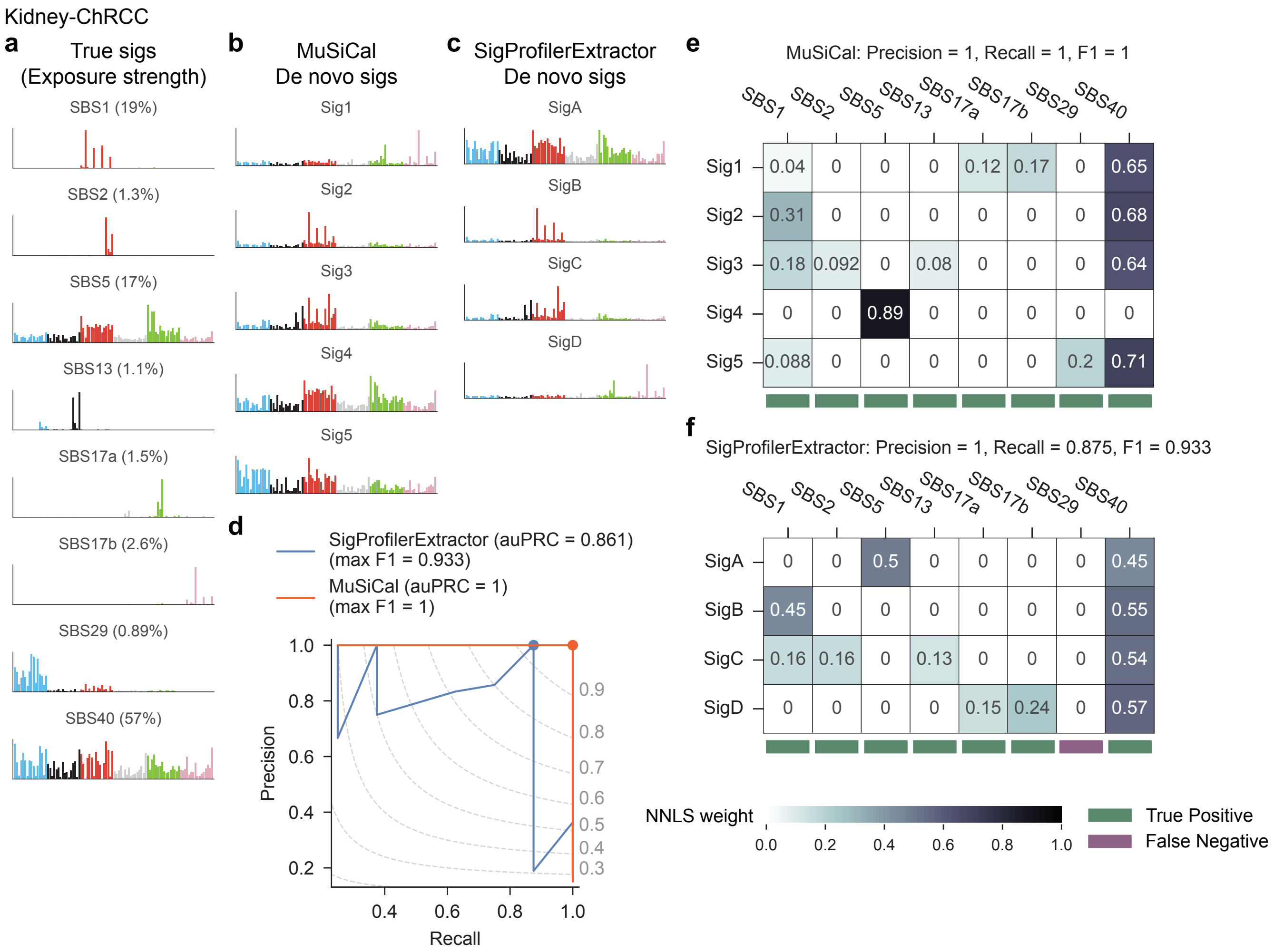

	SBS1	SBS2	SBS5	SBS13	SBS17a	SBS17b	SBS29	SBS40
Sig1	0.04	0	0	0	0.12	0.17	0	0.65
Sig2	0.31	0	0	0	0	0	0	0.68
Sig3	0.18	0.092	0	0.08	0	0	0	0.64
Sig4	0	0	0.89	0	0	0	0	0
Sig5	0.088	0	0	0	0	0	0.2	0.71

	SBS1	SBS2	SBS5	SBS13	SBS17a	SBS17b	SBS29	SBS40
SigA	0	0	0.5	0	0	0	0	0.45
SigB	0.45	0	0	0	0	0	0	0.55
SigC	0.16	0.16	0	0.13	0	0	0	0.54
SigD	0	0	0	0	0.15	0.24	0	0.57

Supplementary Data Fig. 13 Same as Fig. 3a, but for Kidney-ChRCC.

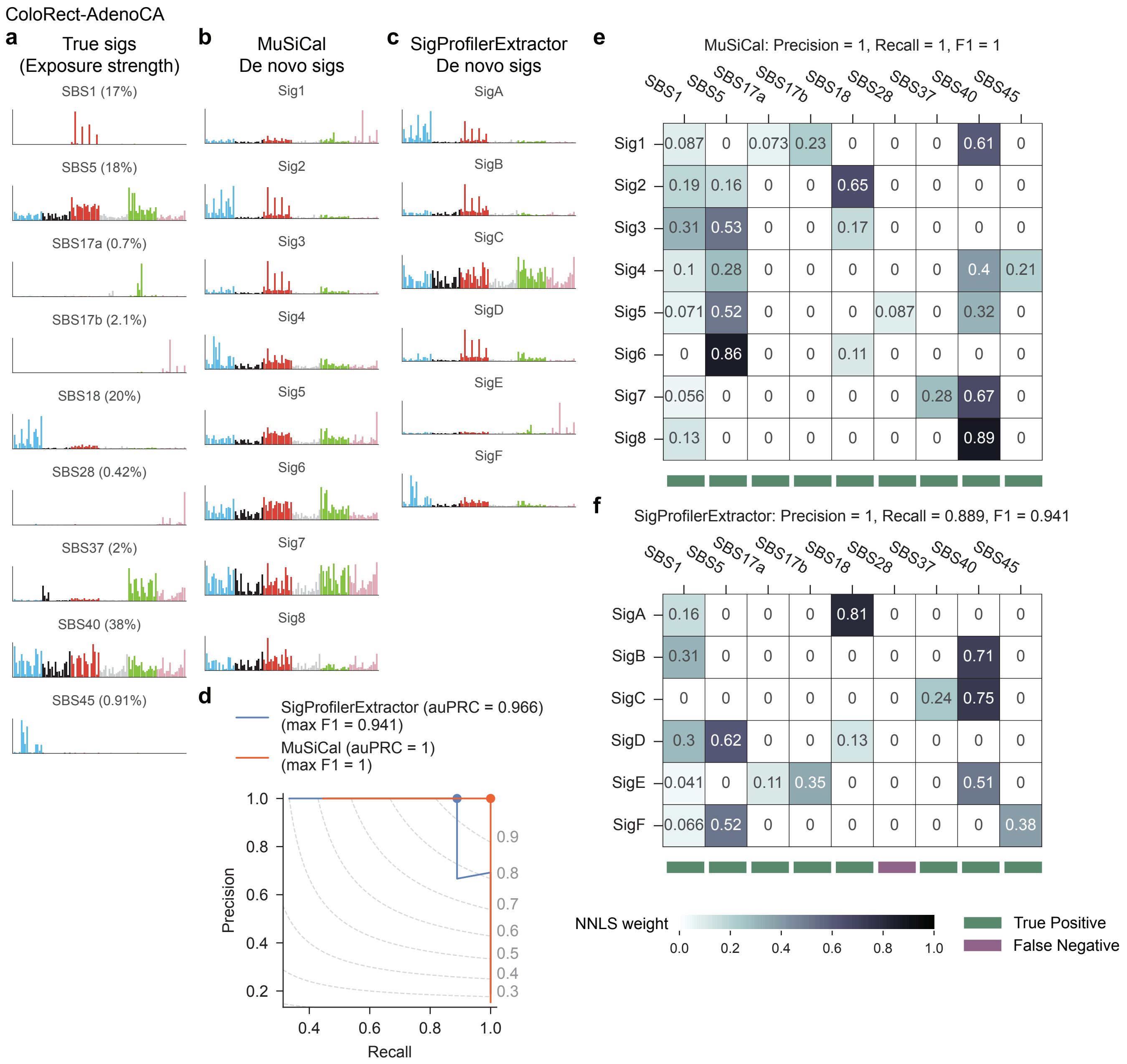

	SBS1	SBS5	SBS17a	SBS17b	SBS18	SBS28	SBS37	SBS40	SBS45
Sig1	0.087	0	0.073	0.23	0	0	0	0.61	0
Sig2	0.19	0.16	0	0	0.65	0	0	0	0
Sig3	0.31	0.53	0	0	0.17	0	0	0	0
Sig4	0.1	0.28	0	0	0	0	0	0.4	0.21
Sig5	0.071	0.52	0	0	0	0.087	0	0.32	0
Sig6	0	0.86	0	0	0.11	0	0	0	0
Sig7	0.056	0	0	0	0	0	0.28	0.67	0
Sig8	0.13	0	0	0	0	0	0	0.89	0

	SBS1	SBS5	SBS17a	SBS17b	SBS18	SBS28	SBS37	SBS40	SBS45
SigA	0.16	0	0	0	0.81	0	0	0	0
SigB	0.31	0	0	0	0	0	0	0.71	0
SigC	0	0	0	0	0	0	0.24	0.75	0
SigD	0.3	0.62	0	0	0.13	0	0	0	0
SigE	0.041	0	0.11	0.35	0	0	0	0.51	0
SigF	0.066	0.52	0	0	0	0	0	0	0.38

Supplementary Data Fig. 14 Same as Fig. 3a, but for ColoRect-AdenoCA.

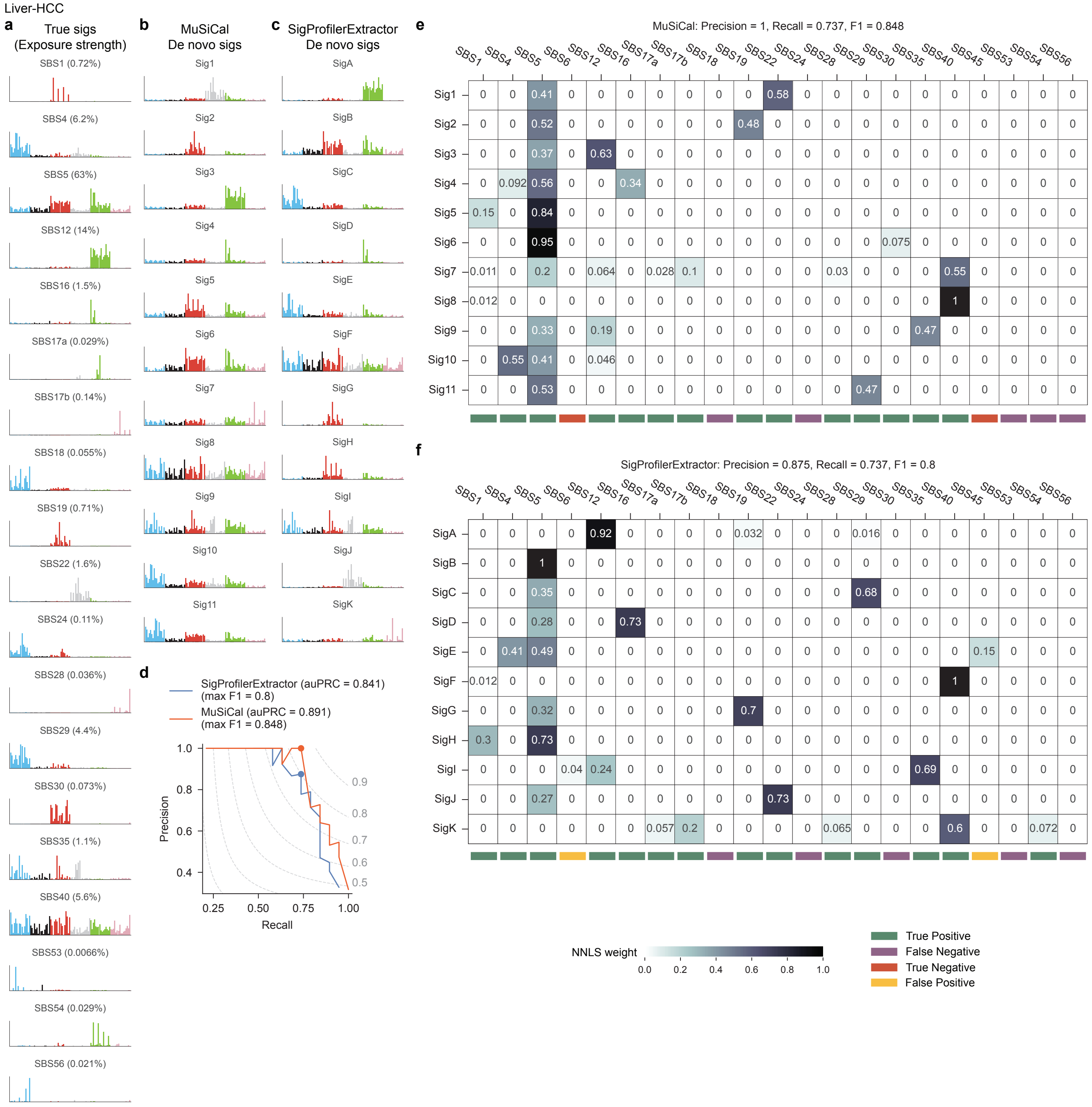

Supplementary Data Fig. 15 Same as Fig. 3a, but for Liver-HCC.

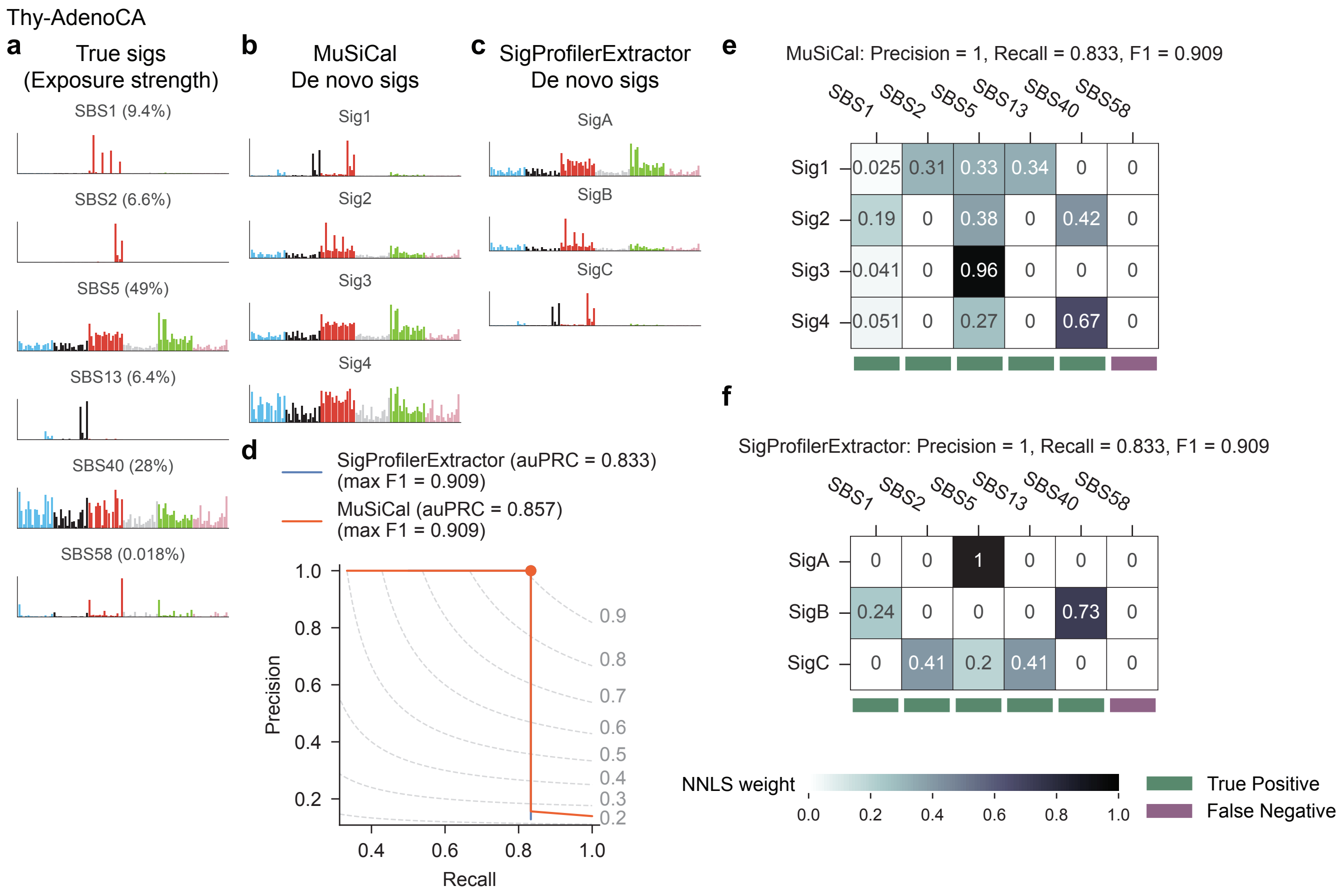

Supplementary Data Fig. 16 Same as Fig. 3a, but for Thy-AdenoCA.

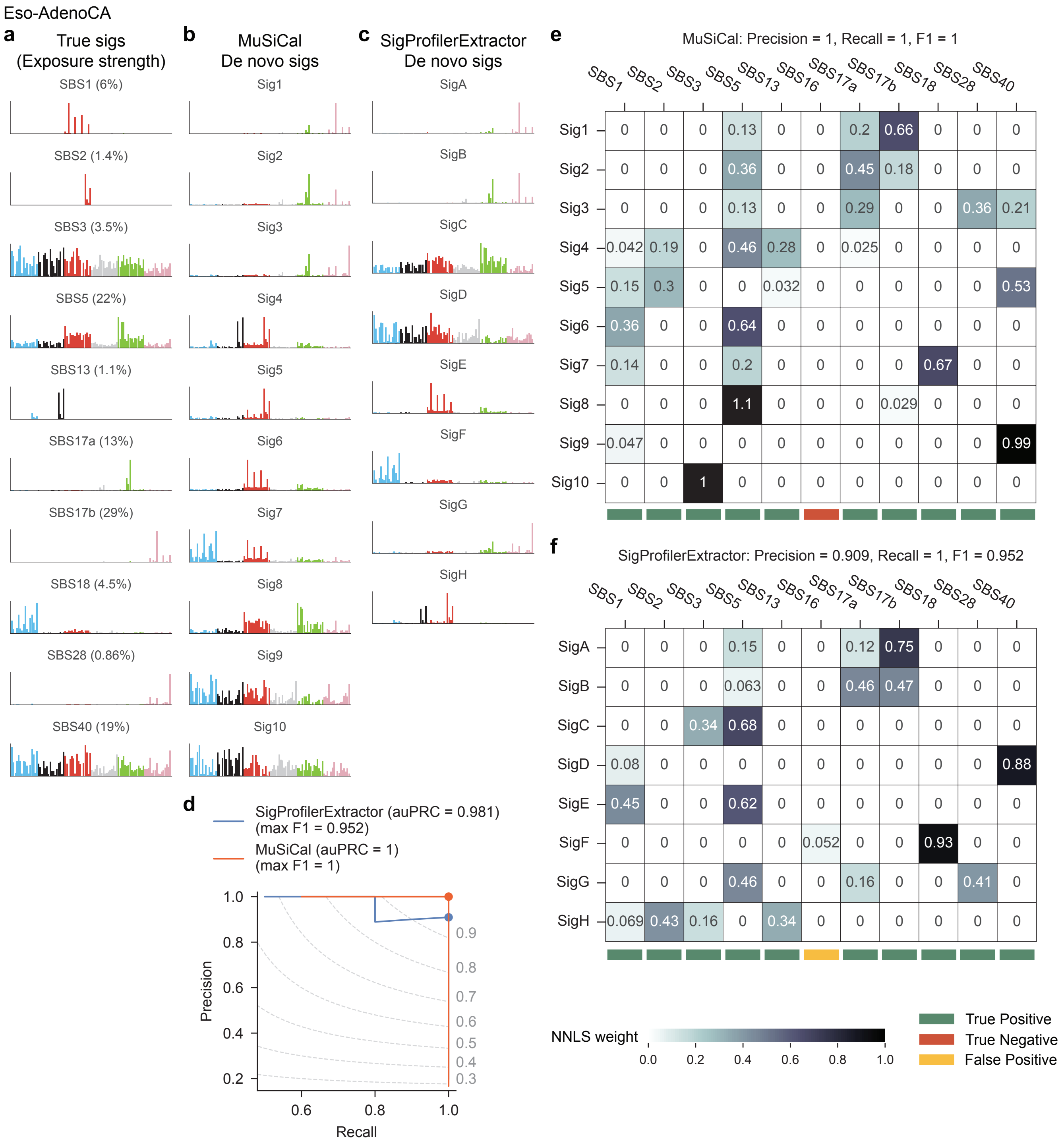

Supplementary Data Fig. 17 Same as Fig. 3a, but for Eso-AdenoCA.

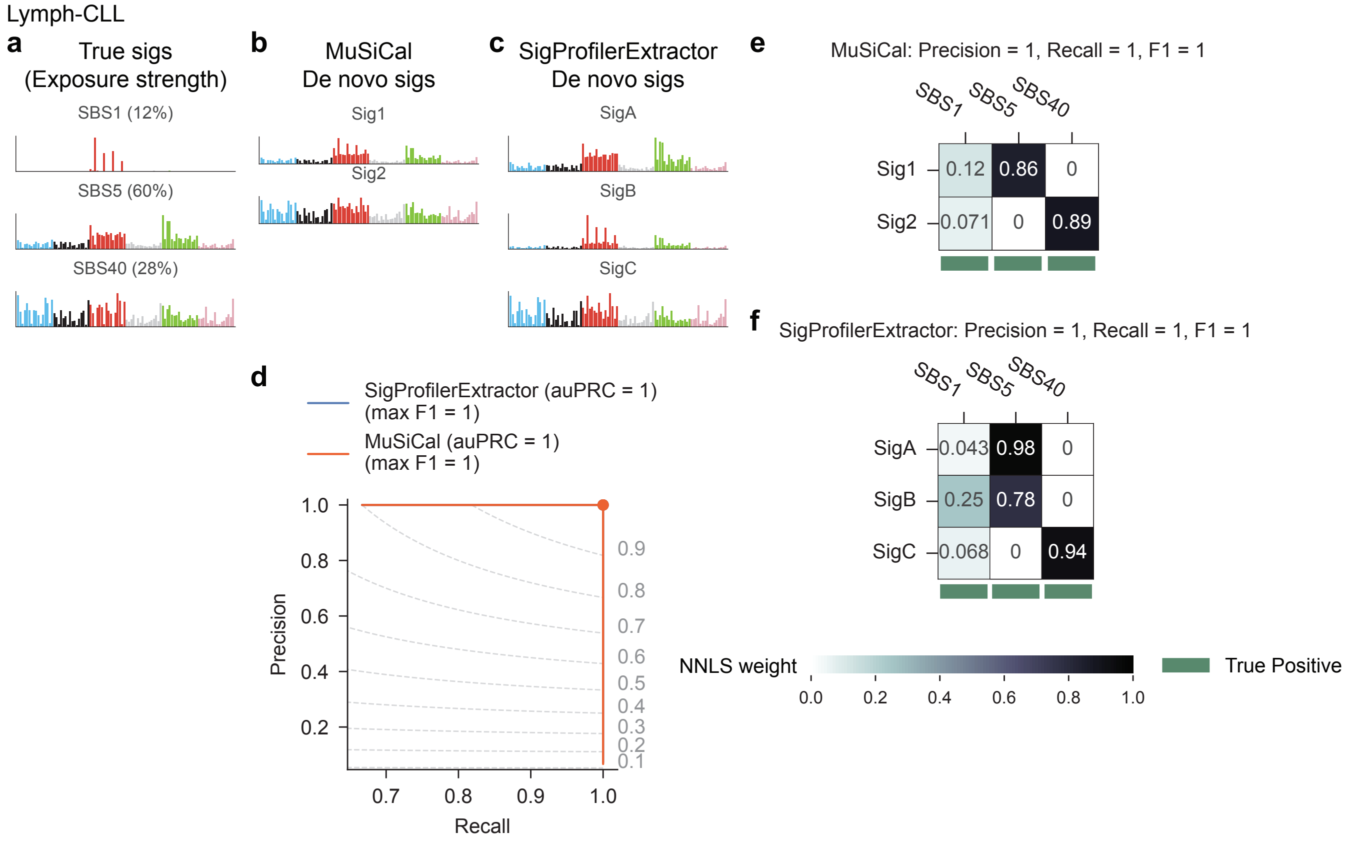

Supplementary Data Fig. 18 Same as Fig. 3a, but for Lymph-CLL.

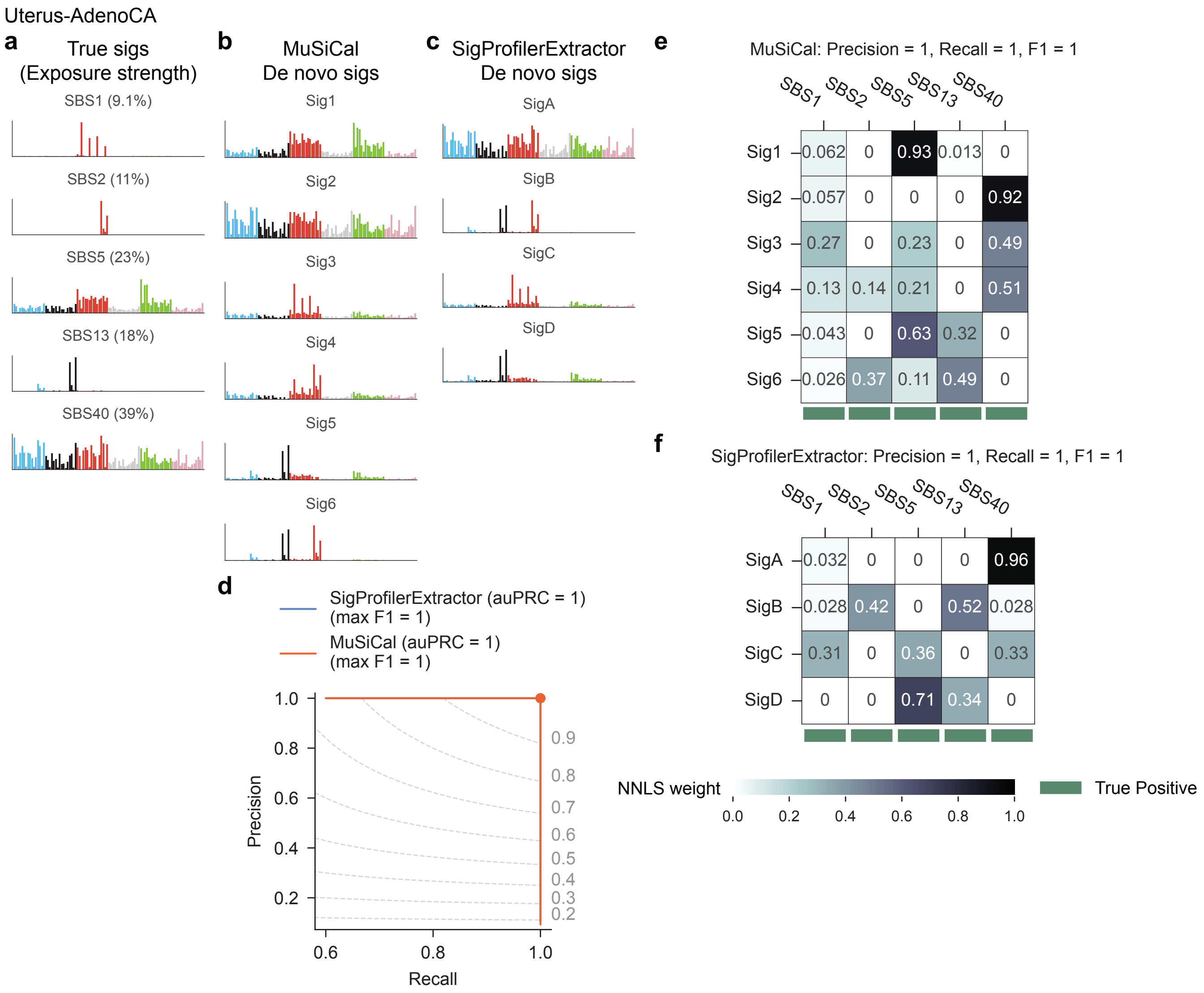

Supplementary Data Fig. 19 Same as Fig. 3a, but for Uterus-AdenoCA.

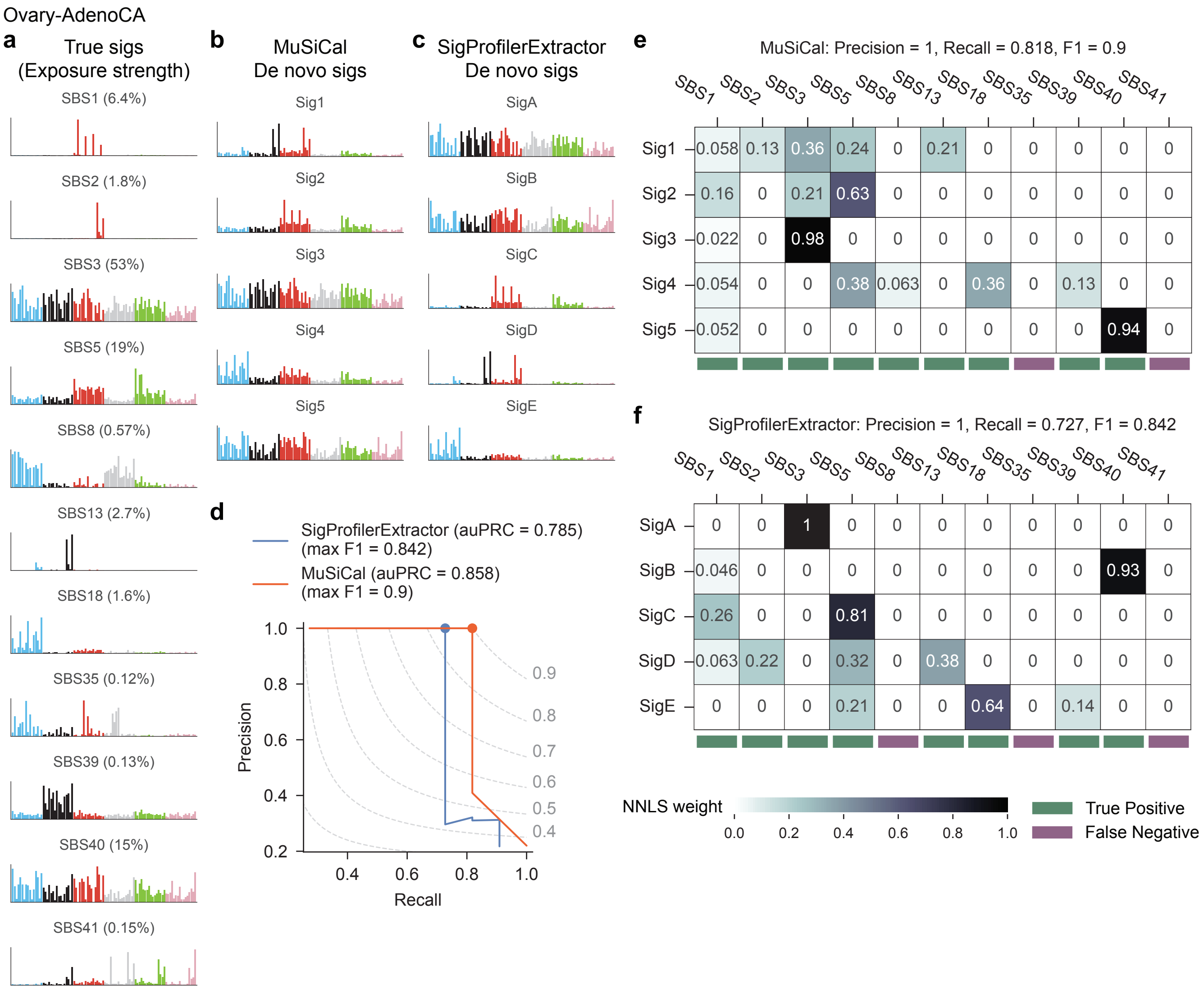

Supplementary Data Fig. 20 Same as Fig. 3a, but for Ovary-AdenoCA.

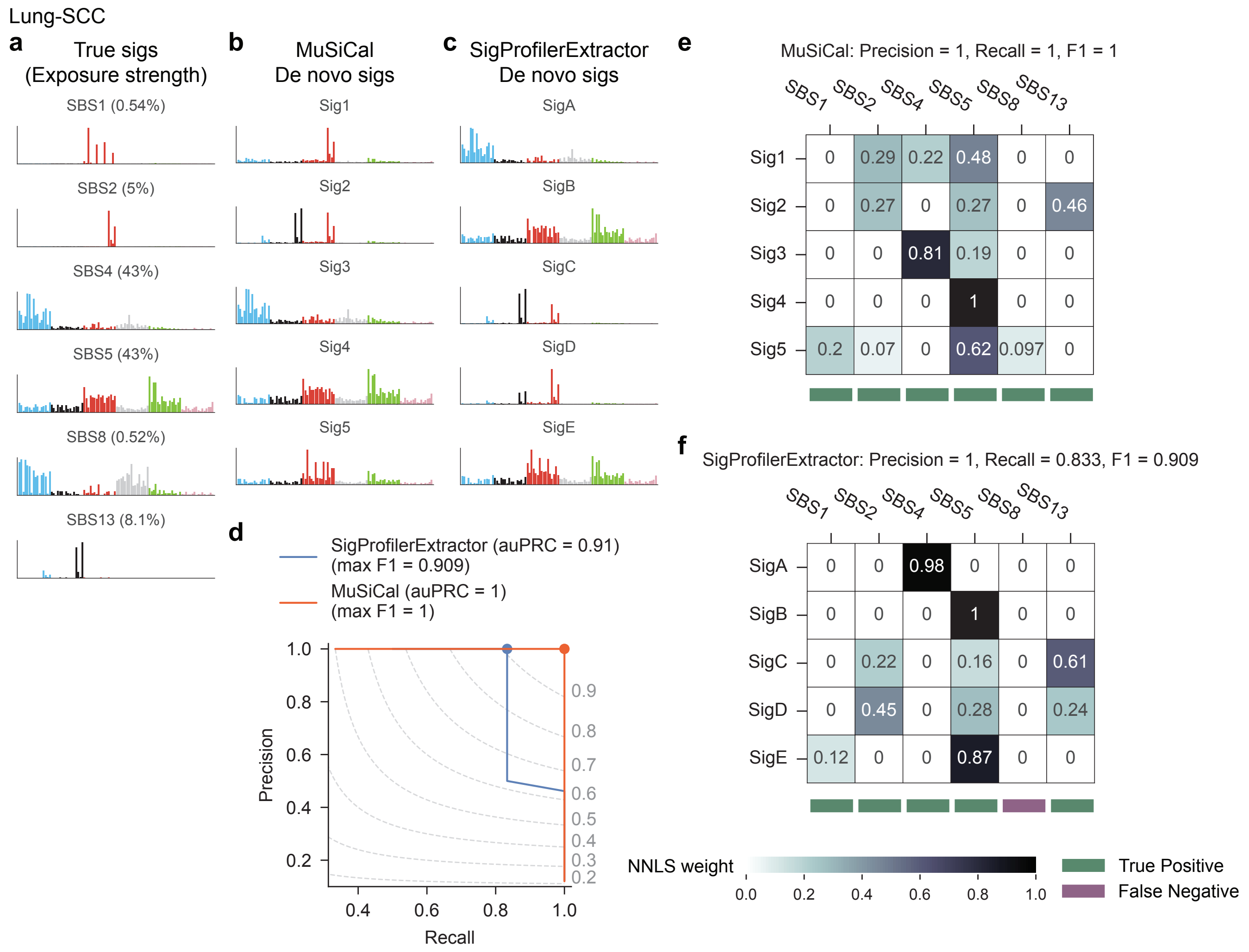

Supplementary Data Fig. 21 Same as Fig. 3a, but for Lung-SCC.

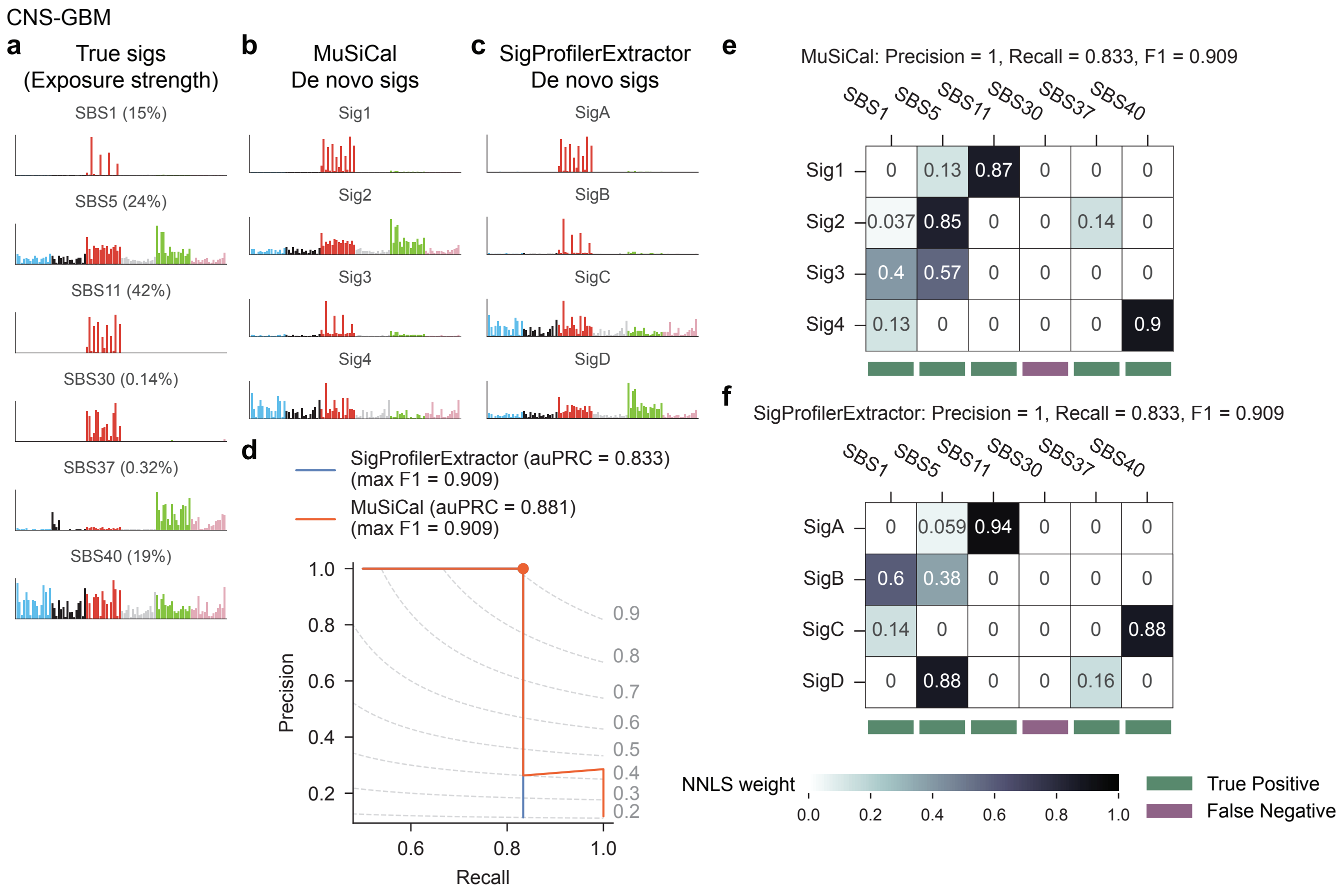

	SBS1	SBS5	SBS11	SBS30	SBS37	SBS40
Sig1	0	0.13	0.87	0	0	0
Sig2	0.037	0.85	0	0	0.14	0
Sig3	0.4	0.57	0	0	0	0
Sig4	0.13	0	0	0	0	0.9

	SBS1	SBS5	SBS11	SBS30	SBS37	SBS40
SigA	0	0.059	0.94	0	0	0
SigB	0.6	0.38	0	0	0	0
SigC	0.14	0	0	0	0	0.88
SigD	0	0.88	0	0	0.16	0

Supplementary Data Fig. 22 Same as Fig. 3a, but for CNS-GBM.

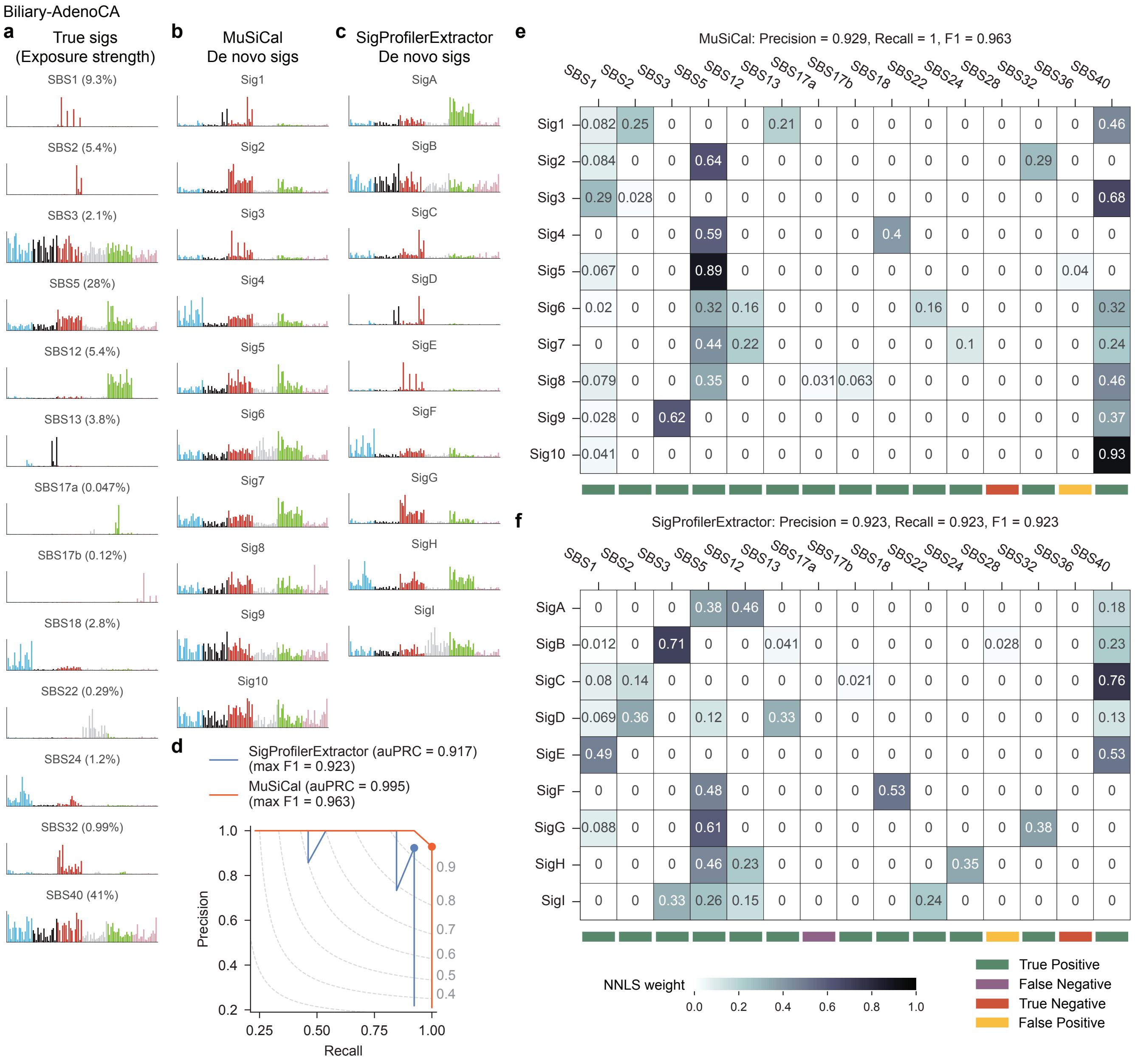

Supplementary Data Fig. 23 Same as Fig. 3a, but for Biliary-AdenoCA.

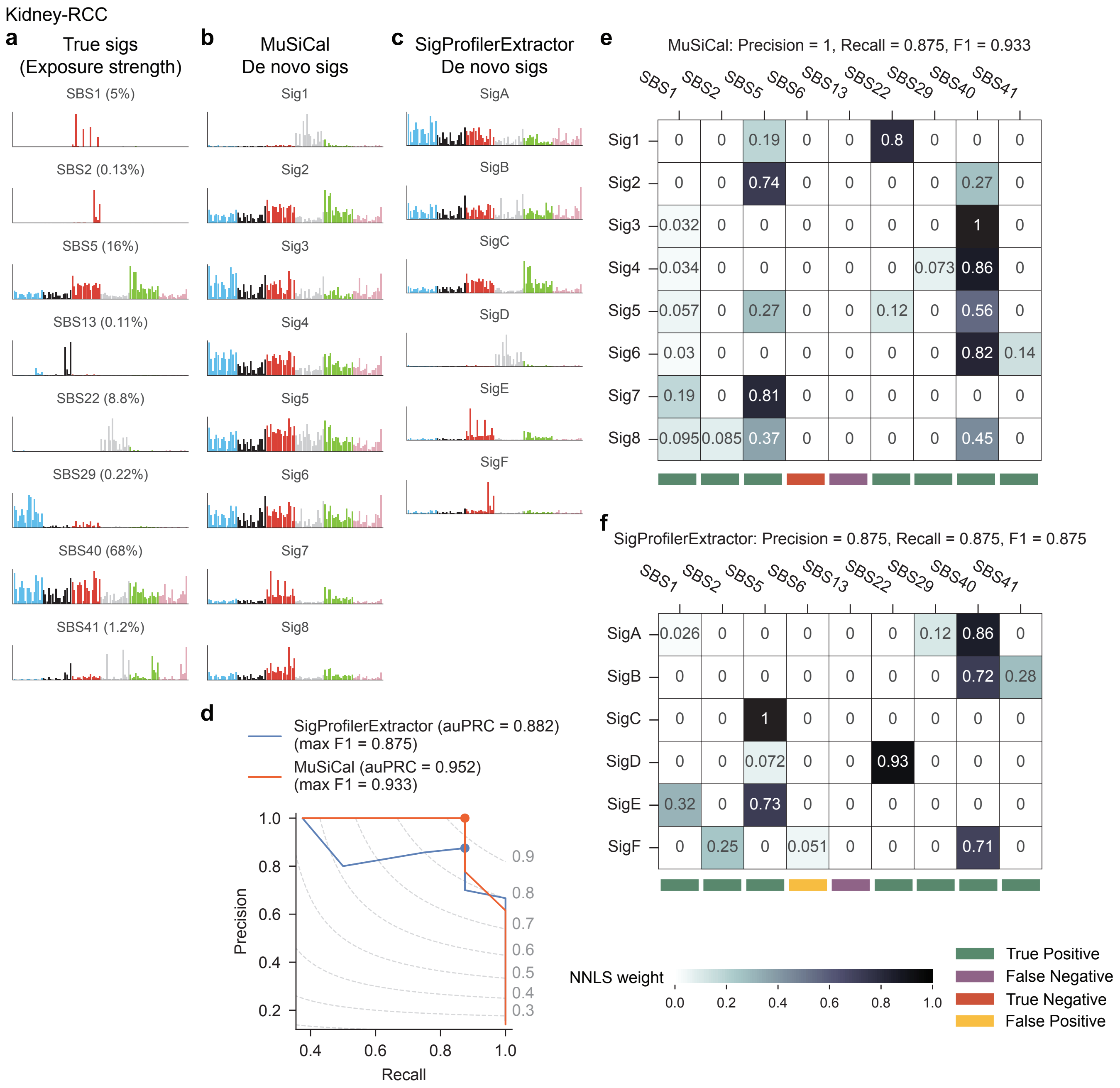

Supplementary Data Fig. 24 Same as Fig. 3a, but for Kidney-RCC.
